# Adaptive (TINT) Changes in the Tumor Bearing Organ Are Related to Prostate Tumor Size and Aggressiveness

**DOI:** 10.1371/journal.pone.0141601

**Published:** 2015-11-04

**Authors:** Hanibal Hani Adamo, Kerstin Strömvall, Maria Nilsson, Sofia Halin Bergström, Anders Bergh

**Affiliations:** Department of Medical Biosciences, Pathology, Umeå University, Umeå, Sweden; Innsbruck Medical University, AUSTRIA

## Abstract

In order to grow, tumors need to induce supportive alterations in the tumor-bearing organ, by us named tumor instructed normal tissue (TINT) changes. We now examined if the nature and magnitude of these responses were related to tumor size and aggressiveness. Three different Dunning rat prostate tumor cells were implanted into the prostate of immune-competent rats; 1) fast growing and metastatic MatLyLu tumor cells 2) fast growing and poorly metastatic AT-1 tumor cells, and 3) slow growing and non-metastatic G tumor cells. All tumor types induced increases in macrophage, mast cell and vascular densities and in vascular cell-proliferation in the tumor-bearing prostate lobe compared to controls. These increases occurred in parallel with tumor growth. The most pronounced and rapid responses were seen in the prostate tissue surrounding MatLyLu tumors. They were, also when small, particularly effective in attracting macrophages and stimulating growth of not only micro-vessels but also small arteries and veins compared to the less aggressive AT-1 and G tumors. The nature and magnitude of tumor-induced changes in the tumor-bearing organ are related to tumor size but also to tumor aggressiveness. These findings, supported by previous observation in patient samples, suggest that one additional way to evaluate prostate tumor aggressiveness could be to monitor its effect on adjacent tissues.

## Introduction

Prostate cancer is a common, generally multifocal, disease with variable behavior ranging from harmless to lethal. Unfortunately our ability to safely diagnose cancer and then differentiate the aggressive from and the non-aggressive forms are limited [1]. Many prostate cancers are difficult to detect by imaging. Tissue biopsies can therefore not be safely guided towards tumors. To circumvent this problem multiple needle biopsies are taken from the organ, but biopsies are small and sample less than 1% of the prostate volume. In most men with raised serum PSA cancer is not found in the biopsies [2–5]. This could be a true or a false negative result. Furthermore when cancer is detected, it is uncertain whether or not the most malignant foci have been sampled. Additional ways to improve prostate cancer diagnostics are therefore needed [2, 3, 5, 6]

Numerous studies have shown that the normal prostate tissue in cancer patients is altered and that detection of such changes (non-malignant prostate tissue is always sampled by the biopsies) can possibly be used to indicate that cancer is present elsewhere in the organ and perhaps also be used to evaluate its future behavior [2, 3, 5, 6] Molecular and microscopic changes in the normal appearing parts of tumor-bearing organs are seen in a variety of tumor types. Such changes are generally considered a result of “field cancerization” [2, 7] By definition the field effect is premalignant epithelial changes induced outside the developing tumor by the carcinogenic agent, although some authors use an extended definition of field effect to include also adaptive changes induced by the tumor [5]. Indeed, changes in the tumor-bearing organ, not restricted to the epithelium but also occurring in the stroma, could be due to signals from the growing tumor. In order to grow and spread neoplastic cells need to influence and interact with adjacent and more remote cells, tissues and organs [8–10]. We have hypothesized that one consequence of this knowledge could be that aggressive cancers, already during early phases of the disease, are able to affect their surroundings in ways quantitatively and qualitatively different than more indolent tumors [3]. If so, increased knowledge about this could be of importance when trying to develop novel ways to diagnose, predicate and treat aggressive prostate cancers.

To explore this possibility in more detail we have implanted locally aggressive rat prostate tumor cells into the prostate of immune-competent syngeneic rats and found that this resulted in adaptive changes in the adjacent normal prostate tissue [4], and that the nature and magnitude of these changes were related to tumor size [11–16]. One change noted in the tumor-bearing prostate was growth of the vasculature, probably necessary to secure the increasing demand of blood supply to and drainage from the growing tumor. Reducing blood flow through the tumor-bearing organ retarded tumor growth [11, 12]. The vascular growth was in part mediated by macrophages[13, 15] and mast cells [14] accumulating in the tumor-bearing organ, particularly in the peri-tumoral region. Depletion of these macrophages [13] and inhibition of the mast cells [14] retarded tumor growth. Similarly, alterations in the non-malignant prostate epithelium [17] and the stroma [3, 6, 14, 16–20] in prostate cancer patients were also related to tumor size and grade, and they could be used to prognosticate the risk of prostate cancer death in a watchful waiting cohort. We have suggested a term for this type of non-malignant tissue; TINT = tumor instructed (and thus indicating) normal tissue [3]. TINT contains morphologically normal appearing epithelium and stroma and is not in direct contact with the cancer epithelium, and should not be confused with the tumor stroma or the tumor-microenvironment.

Several aspects of how the prostate is “tinted” by the presence and nature of a tumor elsewhere in the organ remain unanswered. One important question is if locally aggressive and metastatic cancers influence their surroundings in ways quantitatively or qualitatively different than slow-growing non-metastatic tumor variants. Therefore we implanted poorly differentiated Dunning rat prostate cell lines with different capacities for growth and metastasis into the prostate of immune-competent syngeneic rats. We used the slow growing non-metastatic G variant, the fast growing and locally aggressive but poorly metastatic AT-1, and the fast growing locally aggressive and highly metastatic MatLyLu variant and examined their effects on the tumor bearing- organ. All these cell lines were initially derived from a rat with prostate cancer [21]. In summary we found that TINT-changes were related to tumor size but also to tumor aggressiveness.

## Materials and Methods

### Orthotopic Implantation of Dunning R-3327 Rat AT-1, MatLyLu and G tumor cells

Dunning rat prostate AT-1, MatLyLu and G tumor cells (ATCC, Wesel, Germany) were grown in culture as previously described [4, 11–16].

For morphologic analysis, AT-1 cells (2 x 10^3^ cells in 10 μl of RPMI 1640), MatLyLu (2 x 10^3^ cells in 10 μl of RPMI 1640), or G cells (2 x 10^3^ cells or 2 x 10^5^ cells in 10 μl of RPMI 1640) were carefully injected into one lobe of the ventral prostate of adult Copenhagen rats (Charles River, Sulzfeld, Germany) as previously described [4, 11–16]. Rats were killed at 7 (AT-1, *n* = 5 and MatLyLu, *n* = 8), 10 (AT-1, *n* = 13 and MatLyLu, *n* = 9), 14 (AT-1, *n* = 11), 42 (2 x 10^5^ G cells, *n* = 7) and 49 days (2x10^3^ G-cells, *n* = 6) after tumor cell injection. Rat ventral prostates, injected with RPMI medium, heat-killed tumor cells (100°C, 30 minutes in RPMI), or left intact (non-operated, non-injected) ventral prostate were used as controls. At sacrifice, the animals were injected with bromodeoxyuridine (BrdU, 50 mg/kg body weight; Sigma-Aldrich, Oslo, Norway) to label proliferating cells and pimonidazole (Hypoxyprobe, 60mg/kg body weight; Millipore, MA, USA) to label hypoxic tissue, the prostate tissue was removed, weighed, and prepared as described earlier [11–16].

All of the animal work was approved by the Umeå ethical committee for animal research (permit A110-12) and strong efforts were made to minimize animal discomfort and suffering.

### Tumor Size and Immunohistochemistry

The volume density of tumor tissue was determined on hematoxylin eosin-stained sections as previously described [12]. Total tumor weight was then estimated by multiplying the volume density with prostate weight.

5-μm thick sections were immunostained using primary antibodies against CD68 (AbD Serotec, Oxford, UK), CD163 (AbD Serotec), factor VIII (Dako, Stockholm, Sweden), BrdU (Dako), hypoxyprobe (Millipore) and with toluidine blue as described earlier [11–15].

The volume densities of hypoxyprobe stained prostate epithelium, factorVIII-stained blood vessels, CD68 and CD163 positive macrophages, toluidine blue stained mast cells, and the number of BrdU-labeled endothelial cells per 100 blood vessel profiles (endothelial BrdU labeling), and the number of BrdU labeled vascular mural cells per 100 vascular profiles of non-capillary blood vessels, i.e. small arteries and veins (mural cell BrdU labeling) were measured in the non-malignant parts of the ventral prostate lobe as described earlier [11–15]. As all the histologically normal parts of ventral prostate lobe were included in the measurements the so called TINT values represent an average of the whole non-tumor containing parts of the prostate lobe. The volume densities of macrophages in the tumors and the tumor cell BrdU labeling index were also determined as earlier described [12, 15].

### Statistics

The Mann-Whitney U-test was used for comparison between groups. A *P*-value <0.05 was considered significant. Values represent mean +/- standard deviation. The Spearman rank correlation coefficient (*R*s) was calculated for correlation studies. Statistical analysis was performed using the statistical software Statistica 12.0 (StatSoft, Tulsa, OK, USA).

## Results

### Tumor-Free Prostate Controls

**Fig 1:**
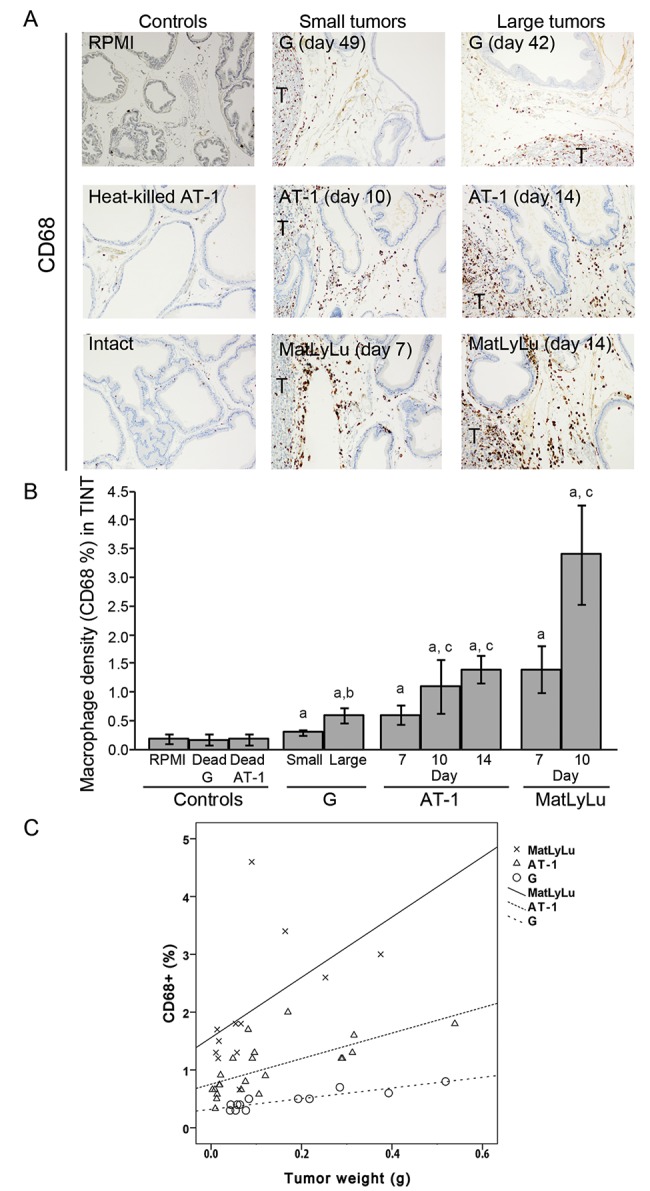
(A) Representative sections from the ventral prostate lobe of Dunning tumor-bearing and control rats stained to visualize CD68^+^ macrophages (brown, 200X magnifications, T; tumor). (B) Volume density of CD68^+^ macrophages in the tumor-adjacent prostate tissue (TINT) and in controls. a; significantly different than controls, b; large G tumors significantly different than small G tumors, and c; significantly different than corresponding tumor at day 7, *P*<0.05, n = 5–13. (C) Scatterplot of the volume density of CD68^+^ macrophages in the tumor-bearing organ plotted against tumor weight (correlation coefficients are given in the result text). The density of macrophages increases with tumor size, but MatLyLu (X) tumors attracted more macrophages than AT-1 (Δ) and G (O). Correlation coefficients are given in results section.

**Fig 2:**
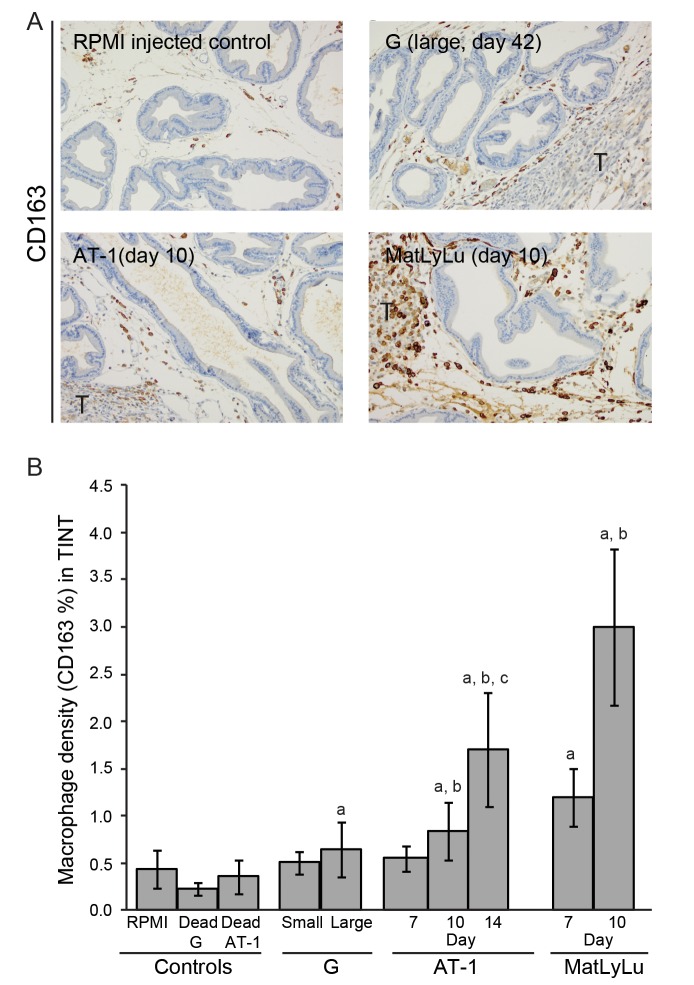
(A) Representative sections showing mainly non-malignant parts of ventral prostate lobe of Dunning tumor-bearing and histologically normal prostate tissue in control rats stained to visualize CD163^+^ macrophages (brown, 200X magnification, the tumor border (marked T) is seen in the periphery of the sections). (B) Volume density of CD163^+^ macrophages in the tumor-adjacent normal prostate tissue (TINT) and in controls. a; significantly different than controls, b; significantly different than corresponding tumor at day 7, and c; significantly different than corresponding tumor at day 10, *P*<0.05, n = 5–13.

**Fig 3:**
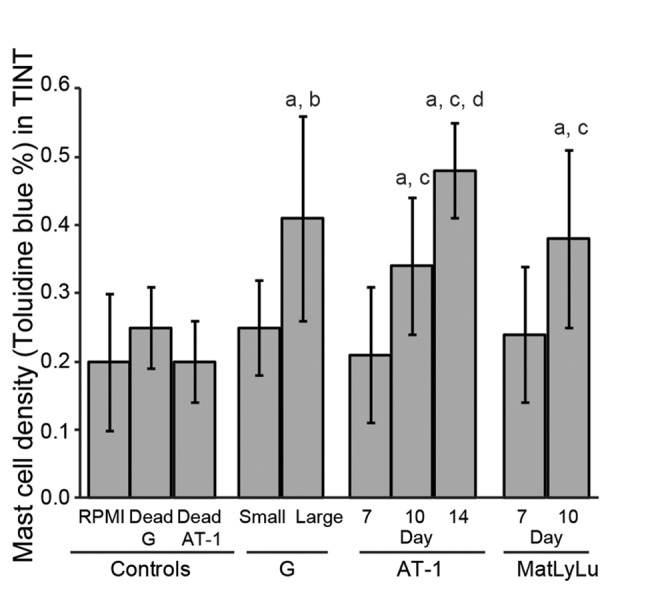
Volume density of toluidine blue^+^ mast cells in the tumor-adjacent prostate tissue (TINT) and in controls, a; significantly different than controls, b; large G tumors significantly different than small G tumors, c; significantly different than corresponding tumor at day 7, and d; significantly different than corresponding tumor at day 10, *P*<0.05, n = 5–13.

**Fig 4:**
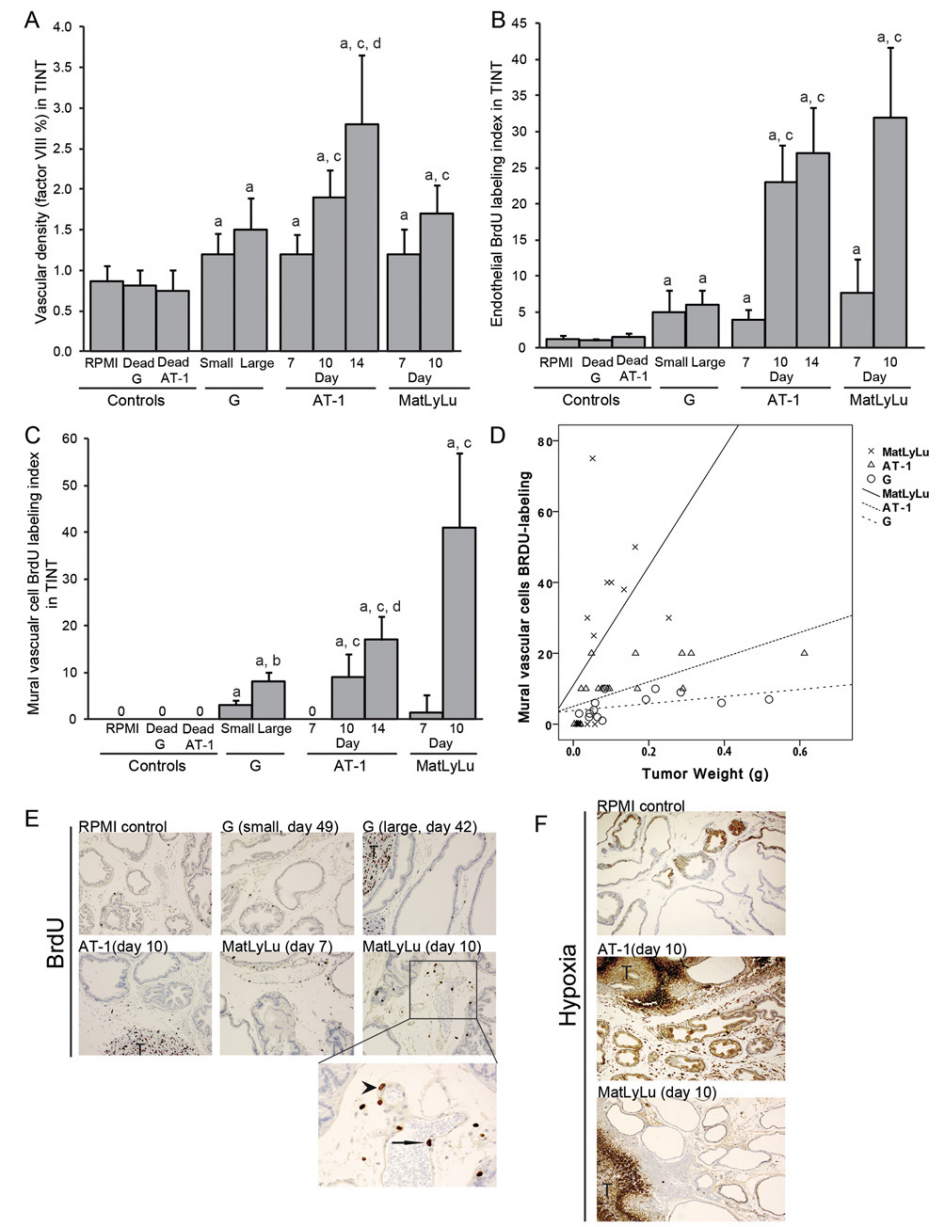
(A) Vascular density (factor VIII), B) endothelial proliferation (BrdU labeling index), and (C) mural vascular cell proliferation (BrdU labeling index) in the tumor-adjacent prostate tissue (TINT) and in controls, a; significantly different than controls, b; large G tumors significantly different than small G tumors, c; significantly different than corresponding tumor at day 7, and d; significantly different than corresponding tumor at day 10, *P*<0.05, n = 5–13. (D) Scatterplot of the BrdU-labeling index in mural vascular cells plotted against tumor size. MatLyLu tumors (X) were considerably more effective than the other tumor types (AT-1; Δ and G; O) in stimulating growth of larger blood vessels in the tumor-bearing organ. Correlation coefficients are given in results section. (E) Representative sections from the ventral prostate lobe of Dunning tumor-bearing and control rats stained to visualize BrdU-labeled cells (Brown, 200X magnifications, T; tumors). Detail (400X magnifications) of vascular (endothelial cell marked with arrow, mural vascular cell marked with arrowhead) BrdU labeling in a 10 day MatLyLu tumor. (F) Representative sections from the ventral prostate lobe of Dunning tumor-bearing and control rats injected with pimonidazole to label hypoxic tissue (brown, 100x magnification, T; tumor).

As injection of vehicle or tumor cells into the prostate likely induces an inflammatory response, it was of importance to evaluate the timing, nature and magnitude of this response. Vehicle (RPMI medium) was injected into the prostate of immune competent rats to examine the response to the injection per se, heat-killed AT-1 or G tumor cells were injected to study the immunogenic response to tumor cell debris. Vascular proliferation and vascular, macrophage and mast cell densities at 10 (RPMI and heat-killed AT-1) and 42 days (heat-killed G) after injection were analyzed in the different control prostates and in intact prostate tissue. No tumors were found in any of the controls and the general morphology of the injected prostate lobes was similar (Fig 1A). Morphology was also similar to that in intact controls (Fig 1A) and to that in the contralateral non-injected prostate lobes (data not shown). Furthermore, there were no significant differences in all values measured between the different controls (Figs 1–4) and compared to intact prostate tissue (data not shown) showing that neither RPMI injection nor injection of heat-killed cells had any large effects on the inflammatory cell infiltration or on angiogenesis.

### Orthotopic Tumor Growth of Different Dunning Rat Prostate Tumor Cells

The Dunning prostate tumors consist of transplantable rat prostatic carcinomas. G, AT-1 and MatLyLu tumors are all poor differentiated tumors but differ in metastatic ability and growth rates [21] We established orthotopic tumors with different tumor sizes from each tumor type, either by following them over time (AT-1 and MatLyLu) or by injecting different number of cells (G) into one ventral prostate lobe. In this way we could examine how tumor size affected the surrounding normal tissue for each tumor type and in addition we could compare TINT changes between the different tumor types by adjusting for size.

When 2x10^3^ was injected, average G tumor size was 49 mg at day 49. When the same number of AT-1 or MatLyLu cells was injected, AT-1 tumor size increased from 15, to 71 and 458 mg from day 7 to day 10 and 14 respectively, and MatLyLu tumor size increased from 35 to 140 mg from day 7 to day 10 (Table 1). This shows that the aggressive MatLyLu tumors at day 7 had almost reached the same size as the slow growing G tumors at day 49 (*P* = 0.27, Table 1). In order to obtain larger G tumors, 2x10^5^ cells were injected. When examined 42 days later, G the mean tumor mass was 250 mg which was roughly of the same size as reached by AT-1 cells after 14 days (*P* = 0.22, Table 1) and MatLyLu cells after 10 days (*P* = 0. 13, Table 1).

**Table 1:** Tumor size and proliferation of different orthotopic Dunning rat prostate tumors.

	G		AT-1			MatLyLu	
Cells injected (n)	2x10^3^	2x10^5^	2x10^3^	2x10^3^	2x10^3^	2x10^3^	2x10^3^
Days after tumor cell injection	49	42	7	10	14	7	10
Animals (n)	6	7	5	13	11	8	9
Tumor weight (mg)	49 +/- 21	250 +/-164*	15 +/-4.5	71 +/-48†	458 +/-406† ‡	35 +/- 24	140+/-111†
Tumor cell proliferation BrdU (%)	23 +/- 3.3	25 +/- 3.4	30 +/- 3.7	30 +/- 4.6	23 +/- 3.9† ‡	41 +/- 8.3	41 +/- 6.6

Values are means +/- SD

* significantly different than G tumors at day 49 (p<0.05)

†significantly different than corresponding tumor at day 7 (p<0.05)

‡ significantly different than the corresponding tumor at day 10 (p<0.05).

Tumor cell proliferation (BrdU) was high in all three tumor types with the highest proliferation seen in the MatLyLu tumors (Table 1). Proliferation in the AT-1 tumors significantly decreased when the tumors were larger at day 14 compared to tumors at day 7 or 10.

### Adaptive Changes of Inflammatory Cells in the Non-Malignant Prostate Tissue Surrounding Rat Tumors

To study the inflammatory cell infiltration, we quantified the average densities of CD68^+^ macrophages (pan macrophage marker, Fig 1), CD163^+^ macrophages (M2, tumor promoting macrophage marker, Fig 2), and toluidine blue^+^ mast cells (Fig 3) in the surrounding tumor bearing prostate tissue (TINT) of all tumor types.

Few CD68^+^ macrophages were observed in the prostate stroma of controls (Fig 1A and 1B) and only some CD68^+^ cells were attracted to the tumor-adjacent prostate tissue of less aggressive G tumors (Fig 1A and 1B) compared to the considerably more macrophages attracted to the tumor-adjacent prostate tissue in animals with aggressive AT-1 and MatLyLu tumors (Fig 1A and 1B). The density of CD68^+^ macrophages in the tumor-adjacent prostate tissue correlated with tumor size for all three tumor-types (G; *R*_s_ = 0.89, *P*<0.05, n = 13, AT-1; *R*_s_ = 0.76, *P*<0.05, n = 29, and MatLyLu; *R*_s_ = 0.69, *P*<0.05, n = 17) (Fig 1B and 1C) but MatLyLu was most effective in stimulating this macrophage infiltration (Fig 1C).

A more pronounced increase of tumor promoting CD163^+^ macrophages was observed in the aggressive AT-1 and MatLyLu tumors compared to the indolent G tumors and compared to controls (Fig 2). Already at day 7, the CD163^+^ macrophage density in prostate tissue surrounding MatLyLu tumors were of the same magnitude as those in prostate tissue surrounding the considerably larger AT-1 tumors at day 10 (Fig 2B). Although the tumors were of similar sizes, prostate tissue in the MatLyLu model had higher CD163^+^ macrophage density at day 7 than prostate tissue in the small G tumors at day 49 (Fig 2B). Tumor weight correlated to volume densities of CD163^+^ macrophages in TINT surrounding G (*R*s = 0.60, p<0.05), AT-1 (*R*s = 0.67, p<0.05) and MatLyLu (*R*s = 0.73 p<0.05) tumors.

The density of mast cells was higher in the tumor bearing prostate tissue of larger tumors compared to control tissue and to tissue surrounding smaller tumors but was generally lower than the density of macrophages (Fig 3). There was no correlation between tumor weight and mast cell density in the prostate tissue surrounding G tumors. In the AT-1 and MatLyLu models, however, tumor weight correlated to the volume density of mast cells (*R*s = 0.75, *R*s = 0.57, respectively, p<0.05).

### Adaptive Changes in Vascular density, Vascular Proliferation and Hypoxia in the Non-Malignant Prostate Tissue Surrounding Rat Prostate Tumors

Vascular growth in TINT is important to supply the growing tumor with adequate blood supply and drainage. We therefore examined vascular density (factor VIII), vascular proliferation (BrdU-labeling index of endothelial and mural vascular cells, and hypoxia in the tumor-bearing organ (Fig 4).

Vascular density in TINT increased gradually with tumor size in all three tumor-types and was higher than in control prostate tissues (Fig 4A). In the aggressive AT-1 and MatLyLu tumors, endothelial proliferation in TINT increased with tumor size while no such effect was seen in the G tumor model (Fig 4B and 4E). No mural cells in larger vessels were proliferating in tumor-free control tissue, but increased considerably with tumor growth in TINT surrounding all tumor types with the highest levels found in the MatLyLu tumor model (Fig 4C–4E). Tumor weight in the AT-1 tumor model was correlated to vascular density (*R*s = 0.53, *P*<0.05), endothelial proliferation (*R*s = 0.69, *P*<0.05), and to mural vascular cell proliferation (*R*s = 0.85, *P*<0.05) in TINT. While in the G and MatLyLu tumor models, tumor weight was only correlated to the mural vascular proliferation (*R*s = 0.63, *R*s = 0.67, respectively, *P*<0.05).

To label hypoxic cells, animals bearing AT-1 tumors, MatLyLu tumors, or RPMI controls were injected with hypoxyprobe prior to sacrifice. As earlier described [22] parts of the glandular epithelium in control ventral prostate tissue was hypoxic (1.8 +/- 1.4%, Fig 4F). In spite of the growth of the prostate vasculature the hypoxic fraction in TINT gradually increased in AT-1 tumor-bearing animals (8.6 +/- 2.8% at day 10 to 13.0 +/- 1.4% at day 14, *P*<0.05, Fig 4F), perhaps explaining the reduced tumor cell proliferation at day 14 (Table 1). In contrast, the hypoxic fraction in the prostate tissue surrounding MatLyLu tumors did not increase with tumor size (3.1 +/- 2.1% at day 7 and 1.7 +/- 0.4% at day 10) and it remained lower than that in prostate tissue surrounding AT-1 tumors at the time-points studied (Fig 4F). The low hypoxia fraction in the tissue surrounding MatLyLu tumors cannot be explained by a larger increase in vascular density in TINT in this model, as the increase in vascular density was similar in both models. MatLyLu tumors were however considerably more effective in stimulating proliferation of mural cells in walls of arterioles and venules in TINT (Fig 4C and 4D) which likely increased the circulation to the tumor and the surrounding non-malignant tissue.

In line with our previous observations in the AT-1 model [13], macrophage density and vascular growth in TINT appeared to be related. For AT-1 tumor-bearing prostates (TINT) the density of CD68+ cells was correlated to micro- and macro-vascular BrdU-labeling (*R*s = 0.82 and *R*s = 0.68, respectively, p<0.05). The respective values for G TINT were *R*s = 0.59 and 0.75 (p<0.05), and for MatLyLu TINT *R*s = 0.82 and *R*s = 0.92 (p<0.05).

### Comparing TINT Changes Surrounding Slow Growing G Compared to the Fast Growing AT-1 Tumors

In order to identify changes more related to tumor type than to tumor size we chose to compare tumors of similar sizes. As the AT-1 mean tumor weight was larger already at day 10 (71 mg) than G tumors at day 49 (49 mg) (Table 1), we deleted the four largest AT-1 tumors from the original data set to obtained groups with similar tumor volumes (Table 2).

**Table 2:** Comparison between G and AT-1 tumors of similar small sizes.

	G tumor	AT-1 tumor
Cells injected (n)	2000	2000
Animals (n)	6	9
Days	49	10
Tumor size (mg)	49 +/- 21	48 +/- 33
Tumor cell proliferation BrdU (%)	23 +/- 3.3	30 +/-3.8 *
CD68 density in tumor (%)	15 +/- 2.9	4.3 +/- 0.9*
CD68 density in TINT (%)	0.34 +/- 0.06	1.0 +/- 0.32*
CD163 density in tumor (%)	0.64 +/- 0.08	0.80 +/- 0.61
CD163 density in TINT (%)	0.51 +/- 0.12	1.1 +/- 0.25*
Mast cell density in TINT (%)	0.25 +/- 0.07	0.37 +/- 0.13
Blood Vessel density in TINT (%)	1.2 +/- 0.20	2.0 +/- 0.32*
Endothelial BrdU labeling in TINT	4.5 +/- 3.1	23 +/- 5.6*
Mural vascular cell BrdU labeling in TINT	2.5 +/- 1.0	8.9 +/- 6.0*

Values are mean+/-SD

* significantly different than in G tumor bearing animals (p<0.05), TINT; Tumor instructed normal tissue (normal prostate tissue in the tumor-bearing organ).

This analysis showed that small AT-1 tumors were more effective than small G tumors in recruiting CD68^+^ and CD163^+^ macrophages, and in stimulating vascular growth, to the tumor-bearing organ (Table 2). Furthermore, we also analyzed the fraction of macrophages infiltrating into the tumor tissue. Compared to the AT-1 tumors, G tumors recruited a substantial number of CD68^+^ macrophages into the tumor (Table 2). These macrophages were, however, mainly CD163^-^ as the density of CD163^+^ macrophages inside the G tumor was low. In contrary to the infiltration in the tumor-bearing organ, the density of tumor infiltrating macrophages was not correlated to tumor size in G or AT-1 tumors.

To explore if these differences were present also around larger tumors we compared AT-1 tumors growing for 14 days with G tumors growing for 42 days. Average tumor sizes for those two groups were 458 vs. 250 mg (Table 1) but by deleting the smallest G, and the three largest AT-1 tumors we obtained groups with similar tumor volumes (Table 3).

**Table 3:** Comparison between G and AT1- tumors of similar large sizes.

	G tumor	AT-1 tumor
Cells injected (n)	200 000	2000
Animals (n)	6	8
Days	42	14
Tumor weight (mg)	282 +/- 154	288 +/- 132
Tumor cell proliferation BrdU (%)	23 +/- 4.1	25 +/- 3.7
CD68 density in tumor (%)	15 +/- 0.89	3.5 +/- 1.4*
CD68 density in TINT (%)	0.60 +/- 0.13	1.4 +/- 0.24*
CD163 density in tumor (%)	0.54 +/- 0.26	0.66 +/- 0.30
CD163 density in TINT (%)	0.69 +/- 0.30	1.9 +/- 0.62*
Mast cell density in TINT (%)	0.42 +/- 0.16	0.48 +/- 0.08
Blood vessel density in TINT (%)	1.5 +/- 0.42	2.8 +/- 1.0*
Endothelial BrdU labeling in TINT	6.0 +/- 2.1	27 +/- 6.3*
Mural vascular cell BrdU labeling in TINT	8.2 +/- 1.7	16 +/- 5.5*

Values are mean+/-SD

* significantly different than in G tumor bearing animals (p<0.05), TINT; Tumor instructed normal tissue (normal prostate tissue in the tumor-bearing organ).

This analysis showed that also large AT-1 tumors had a higher capacity to attract more CD68^+^ and CD163^+^ macrophages, and to stimulate vascular growth in the surrounding normal prostate tissue than large G tumors (Table 3).

### Comparing TINT Changes Surrounding Low-Metastatic AT-1 and Highly Metastatic MatLyLu Tumors

Ten days after tumor cell injection, average tumor weight was 2-fold larger in MatLyLu (140mg) than in AT-1 tumors (71mg) (Table 1), although the difference was not statistically significant, suggesting that MaLyLu in average grows faster than AT-1. A view supported by a higher tumor cell BrdU labeling index in MatLyLu than in AT-1 tumors (Table 1).

In order to identify changes in the surrounding prostate tissue that are more related to tumor type than to tumor size we deleted the five smallest AT-1 and the two largest MatLyLu tumors from the original dataset (Table 4). When AT-1 and MatLyLu tumors of similar sizes were compared it was apparent that MatLyLu tumors were more successful in attracting macrophages, stimulating growth of larger blood vessels and limiting hypoxia in the tumor-bearing organ (Table 4).

**Table 4:** Comparison between AT-1 and MatLyLu tumors of similar sizes.

	AT-1	MatLyLu
Cells injected (n)	2000	2000
Animals (n)	8	7
Days	10	10
Tumor weight (mg)	97 +/- 39	90 +/- 47
Tumor cell proliferation BrdU (%)	29 +/- 4.5	42 +/- 7.3*
CD68 density in tumor (%)	4.3 +/- 0.3	9.9 +/- 1.5*
CD68 density in TINT (%)	1.2 +/- 0.5	3.3 +/- 1.4*
CD163 density in tumor (%)	0.8 +/- 0.6	2.3 +/- 1.2*
CD163 density in TINT (%)	1.0 +/- 0.22	2.8 +/- 0.59*
Mast cell density in TINT (%)	0.30 +/- 0.07	0.34 +/- 0.12
Blood vessel density in TINT (%)	1.9 +/- 0.37	1.7 +/- 0.40
Endothelial BrdU labeling in TINT	23 +/- 4.5	32 +/- 11
Mural cell BrdU labeling in TINT	10 +/- 0.1	42 +/- 16*
Hypoxyprobe stained TINT (%)	7.4 +/- 2.0	3.9 +/- 1.6*

Values are mean+/-SD

* significantly different than in AT-1 tumor bearing animals (p<0.05), TINT; Tumor instructed normal tissue (normal prostate tissue in the tumor-bearing organ).

### Comparing TINT changes in the rat prostate with alterations found in non-malignant parts of the prostate in prostate cancer patients

In the rat model some tumor-induced changes in the tumor-bearing organ were related to tumor size whereas others were related also to tumor aggressiveness (growth rate and metastatic capacity). How does this pattern compare to finding in patients? We have, by analyzing a large historical cohort of patients with voiding symptoms diagnosed with prostate cancer after transurethral resection of the prostate (TUR-P) and managed with watchful waiting, previously described a number of morphological changes in the non-malignant parts of the tumor-bearing organ related to patient outcome. These changes (presumably TINT and/or field effects) and their relations to tumor Gleason score, clinical tumor stage and estimated tumor size (percentage of resected tissue containing tumor) are now summarized and categorized in Table 5. Some alterations in the non-malignant parts of the prostates (associated with outcome) were related both to tumor aggressiveness (Gleason score) and to tumor size (clinical stage, and estimated tumor size). Other changes in the tumor-bearing human prostate tissue of prognostic significance were apparently unrelated both to tumor clinical stage and size, or only related to tumor Gleason score. No changes of prognostic significance were related only to tumor size.

**Table 5:** Summary of previously reported TINT factors in human prostate cancer patients that relate to patient outcome.

Factor in TINT	Tumor Gleason score*	Tumor clinical stage*	Estimated tumor size*
Epithelial pAKT [19]	R = 0.13, n = 240, p = 0.04	R = 0.14, n = 238, p = 0.03	R = 0.17, n = 240, p = 0.007
Stroma PDGFR-beta [23]	R = 0.15, n = 355, p = 0.004	R = 0.11, n = 349, p = 0.033	R = 0.16, n = 355, p = 0.003
Epithelial LRIG1 [24]	R = 0.18, n = 191,p = 0.014	R = 0.14, n = 190, p = 0.049	R = 0.20, n = 191, p = 0.006
Epithelial pEGFR [17]	R = 0.26, n = 174, p = 0.000	Non-correlated	Non-correlated
Stroma Mast cells [14]	R = 0.15, n = 358, p = 0.01	Non-correlated	Non-correlated
Stroma Hyaluronan [16]	R = 0.21, n = 216, p = 0.002	Non-correlated	Non-correlated
Stroma CD163+ macrophages [25]	R = 0.19, n = 105, p = 0.049	Non-correlated	Non-correlated
Stroma blood vessels [26]	Non-correlated	Non-correlated	Non-correlated
Stroma S100A9+ macrophages [20]	Non-correlated	Non-correlated	Non-correlated

*Association (R, Spearman rho) with TINT factor (n = number of patients examined).

## Discussion

Studies during the last decades have shown that tumors, in order to growth and spread, have to interact significantly with both adjacent stroma cells within the tumor (the tumor microenvironment) and with remote organs. This interaction is accomplished by secretion of both paracrine and long-ranging signals that instruct adjacent stroma cells and other organs, such as the bone-marrow and pre-metastatic niches, to prepare the soil for subsequent growth, spread and metastatic colonization, but also to attract cells from other organs to the growing tumor mass [8–10, 27, 28]. One site, considerably less studied, that probably also have to adapt to the needs of the growing tumor is substantial parts of the tumor-bearing organ [3].

The aim of this study was to explore in more detail if and how the tumor-bearing organ is influenced by the growth of prostate tumors of different aggressiveness. To explore this we used an animal model where poorly differentiated rat prostate cancer cells, all initially defied form a single rat prostate tumor [21], but with different growth and metastatic potentials were injected into the prostate of immune-competent rats.

As we injected presumably antigenic cells into fully immune-competent syngeneic animals this could induce an immune response that could be unspecific to the presence of a growing tumor and to tumor characteristics. We therefore first examined the magnitude of the host response in prostates injected with only vehicle or with heat-killed tumor cells. We conclude that the response to vehicle is very discrete and similar to that of heat-killed tumor cells and that these responses are of considerably lower magnitude than those induced by growing tumor cells. Furthermore, we show that small but highly aggressive and metastatic tumor cells were able to induce a host response in the tumor-bearing organ of considerably higher magnitude than that of poorly metastatic or much larger but more slowly growing tumors. We therefore consider that our model is suitable to experimentally explore how tumors with different malignant potential affect the tumor-bearing organ, and the functional role of these changes.

A useful experimental model should, at least in some important aspects, mimic the situation in patients. To some extent this appears to be the case as there are more pronounced changes in the tumor-bearing organ (measured at random distances form tumor), carrying high-grade tumors with poor outcome than in cases carrying indolent low-grade prostate tumors (Table 5) [3, 6] As suspected there is not a perfect match between the factors now measured in the rat model and the corresponding factors in human samples. In patients the density of tumor promoting macrophages [20], mast cells [14], and blood vessels [26] are higher in prostate tissue surrounding aggressive vs. non-aggressive prostate cancers, and they can be used to predict outcome. In patients the relations of these three factors to tumor-type and tumor size were not as strong as in the animal model possibly because in patients they are affected also by other factors not present in a pure model. Nevertheless, the rat models and the corresponding findings in patients suggest that the tumor-containing prostate is differently “tinted” by aggressive vs. non-aggressive tumors. The host response to a tumor in experimental models and in patients is strikingly similar to a wound-response [29–32], and the global gene-expression pattern in the tumor and importantly also in the non-malignant part of an AT-1 bearing prostate lobe resembled a wounding-response [4]. Tumors apparently exploit their capacity to induce an inflammatory reaction in the tumor microenvironment extending out into the tumor-bearing organ (the TINT) by secreting factors that attract and reeducate the accumulating inflammatory cells to support tumor growth [9, 10, 33, 34]. The exact nature of the tumor-derived signals inducing TINT is unknown, but factors attracting and regulating macrophages are among the likely candidates [4, 13].

The fast growing locally aggressive and metastatic MatLyLu tumors appear to be particularly effective in recruiting macrophages, of which many are of the tumor-stimulating M2 phenotype (CD163 positive), to the tumor, to the invasive front and to the surrounding tumor-bearing organ. Depletion of these macrophages reduced tumor growth, vascular densities and micro- and macro-vascular BrdU labeling in prostates implanted with AT-1 tumors [13, 15]. It is therefore likely that these macrophages stimulate vascular and tumor growth, and could be involved in the tissue reorganization processes necessary for subsequent growth and spread also of MatLyLu tumors. One interesting difference between the metastatic MatLyLu tumor and the fast growing but poorly metastatic AT-1 tumor is that MatLyLu tumors appear to be particularly effective in stimulating growth (increasing BrdU labeling among mural vascular cells) of larger blood vessels. To secure sufficient blood supply to an expanding tumor, growth of arteries and veins in the tumor-bearing organ, is probably as important as proliferation of endothelial cells in the tumor micro-vasculature [13, 35]. In other tissues, growth of arteries and veins is mediated by macrophages, in particular of the M2 phenotype [36–39]. A functionally more effective vascular system could perhaps explain why the hypoxic fraction of the tumor bearing-organ is lower in the MatLyLu than in the AT-1 model. Future studies in patient samples should examine whether signs of macro-vascular growth in the tumor-bearing prostate could be used to evaluate tumor aggressiveness.

In this study we examined the MaLyLu model up to 10 days after tumor cell implantation. At this time-point metastases cannot be detected in lymph nodes or in lungs but such metastases can be seen one week later (own unpublished observations). It is therefore possible that the requirements for metastatic seed of tumor cells are prepared for already at 10 days. One cell-type involved in facilitating vascular invasion, extravasation and colonization of tumor cells is macrophages [9, 40]. Future studies should therefore examine whether metastasis promoting macrophage subtypes [40–42] are particularly abundant in the MatLyLu model.

Mast cells were attracted to the prostate tissue surrounding Dunning tumors, and inhibition of mast cells function retarded vascular and tumor growth in the AT-1 model [14]. Mast cell densities in the tumor-bearing organ however appeared to be more closely related to tumor size than to tumor type when comparing the metastatic MatLyLu to the AT-1 model. This may suggest that mast cells could be more involved in mediating growth than in creating the specific micro-environmental changes necessary for subsequent metastasis.

In summary, in this paper we demonstrate that implantation of tumor cells into the rat prostate results in adaptive, presumably tumor promoting, changes in the tumor-bearing organ. Some of these adaptive TINT changes appear to be mainly related to the tumor size whereas others are also related to the growth and metastatic potential of the growing tumor. Several investigators have already described changes in the tumor-bearing human prostate related to the presence, and for some factors also to the grade and distance to tumors present elsewhere in the organ [2, 3, 5, 6] Such changes have generally been explained as a result of field cancerization (i.e. precancerous epithelial changes occurring outside the tumor) [2, 5] In human prostate tissue, cancer field-effects, adaptive TINT-effects, as well as effects of concomitant pathologies such as prostatitis and benign hyperplasia could all be involved in reshaping the tumor-bearing organ. Our rat model can be used to pinpoint and explain the mechanisms behind the growth and metastasis promoting changes that some tumors can induce in a previously normal organ (allowing the separation of TINT effects from effects of field cancerization). Further studies are needed to define these changes and to explore their potential utility as additional diagnostic and prognostic markers as well as novel therapeutic targets.
